# Hospital Crests and Badges

**Published:** 1911-12-02

**Authors:** J. Wilson

**Affiliations:** Vice-President Midwives Institute. Albemarle Club, W.


HOSPITAL CRESTS AND BADGES.

*To the Editor of* The Hospital.

Sir,—I am making a collection of the coats of arms, crests, and badges of all our hospitals in the United Kingdom. I should be grateful if you would kindly print this letter, as some secretaries may, on reading it, kindly help me. I would like the pattern to be not too small, and if uncoloured to have the proper colours written at the side. My object is a large piece of needlework—probably in the form of a banner—which will represent in this form what our older hospitals have done and are doing for suffering humanity. I expect to include special hospitals of standing in the United Kingdom, and shall be most grateful for the needful help.

Truly yours,

J. Wilson, Vice-President Midwives Institute.

Albemarle Club, W.

November 26.

